# Effects of immunostimulation on social behavior, chemical communication and genome-wide gene expression in honey bee workers (*Apis mellifera*)

**DOI:** 10.1186/1471-2164-13-558

**Published:** 2012-10-16

**Authors:** Freddie-Jeanne Richard, Holly L Holt, Christina M Grozinger

**Affiliations:** 1 Laboratoire Ecologie Evolution Symbiose, UMR CNRS 6556, University of Poitiers, 40 avenue du Recteur Pineau, Cedex, F-86022, POITIERS, France; 2 Department of Entomology, Center for Pollinator Research, Center for Chemical Ecology, Huck Institutes of the Life Sciences, Pennsylvania State University, University Park, PA, 16802, USA; 3 Previous address: Department of Entomology, North Carolina State University, Raleigh, NC, 27695, USA

**Keywords:** Honey bees, Immunity, Genomics, Social behavior, Social immunity, Chemical ecology, Cuticular hydrocarbons, Nestmate recognition

## Abstract

**Background:**

Social insects, such as honey bees, use molecular, physiological and behavioral responses to combat pathogens and parasites. The honey bee genome contains all of the canonical insect immune response pathways, and several studies have demonstrated that pathogens can activate expression of immune effectors. Honey bees also use behavioral responses, termed social immunity, to collectively defend their hives from pathogens and parasites. These responses include hygienic behavior (where workers remove diseased brood) and allo-grooming (where workers remove ectoparasites from nestmates). We have previously demonstrated that immunostimulation causes changes in the cuticular hydrocarbon profiles of workers, which results in altered worker-worker social interactions. Thus, cuticular hydrocarbons may enable workers to identify sick nestmates, and adjust their behavior in response. Here, we test the specificity of behavioral, chemical and genomic responses to immunostimulation by challenging workers with a panel of different immune stimulants (saline, Sephadex beads and Gram-negative bacteria *E. coli*).

**Results:**

While only bacteria-injected bees elicited altered behavioral responses from healthy nestmates compared to controls, all treatments resulted in significant changes in cuticular hydrocarbon profiles. Immunostimulation caused significant changes in expression of hundreds of genes, the majority of which have not been identified as members of the canonical immune response pathways. Furthermore, several new candidate genes that may play a role in cuticular hydrocarbon biosynthesis were identified. Effects of immune challenge expression of several genes involved in immune response, cuticular hydrocarbon biosynthesis, and the Notch signaling pathway were confirmed using quantitative real-time PCR. Finally, we identified common genes regulated by pathogen challenge in honey bees and other insects.

**Conclusions:**

These results demonstrate that honey bee genomic responses to immunostimulation are substantially broader than the previously identified canonical immune response pathways, and may mediate the behavioral changes associated with social immunity by orchestrating changes in chemical signaling. These studies lay the groundwork for future research into the genomic responses of honey bees to native honey bee parasites and pathogens.

## Background

Honey bees are an outstanding model system for studying the molecular, physiological and social basis of disease transmission and resistance. Honey bees are plagued by a number of parasites and pathogens (reviewed in [1]), and the social colony environment (with up to 50,000 densely packed worker bees [2]) provides excellent conditions for disease transmission. While the innate immune systems of invertebrates and vertebrates are surprisingly conserved [3], there can be large differences in the numbers of genes involved in the different molecular arms of the immune response system across species. As is the case with many insects with recently sequenced genomes [4-8], bees have a much smaller number of known canonical immune response genes relative to *Drosophila*[9], which is one of the best characterized models of insect immunity [10]. Thus, bees (and other insects) may utilize alternative genetic and physiological mechanisms to respond to infections. Furthermore, honey bees can resist pathogens and parasites by employing sophisticated behavioral defense mechanisms, termed “social immunity” (reviewed in [11]). Here, we examine the effects of a panel of general immune elicitors (injection with saline, Sephadex beads or bacteria) on worker-worker social interactions and chemical communication, and use whole-genome microarrays to characterize global gene expression responses to these immune elicitors.

Honey bee populations have been in decline worldwide, with beekeepers recently reporting massive annual losses (>30%) (reviewed in [1]). This decline is undoubtedly due in part to the multitude of parasites and pathogens that target honey bees, several of which have only recently been identified. Honey bees are host to over 20 viruses [12-14], as well as a number of bacterial and fungal pathogens [15-18] including the gut microspordian parasites *Nosema apis* and *Nosema ceranae*. Honey bees are also severely impacted by *Varroa* mites (*Varroa destructor*), tracheal mites (*Acarapis woodi*) and other ectoparasites [19]).

Sequencing of the honey bee genome identified 177 genes associated with the canonical immune response pathways in insects [9]. Innate immune pathways in insects, mostly obtained from studies in *Drosophila*, consists of both cellular and humoral responses, which can be systemic or local (reviewed in [10,20-24]). Cellular immune responses involve a number of differentiated hemocytes. Pathogens activate phenoloxidase and associated immune cascades, resulting in phagocytosis, encapsulation, and/or melanization of invading organisms or wounds. Humoral responses include cytotoxic molecules (such as reactive oxygen and nitrogen species), lysozymes, cytokines, and antimicrobial peptides (AMPs). AMPs are primarily produced by the fat bodies in insects. A number of signal transduction pathways are involved in moderating immune responses, including the JAK/STAT (which seems to respond primarily to tissue damage), JNK (which mediates wound repair), Imd (which primarily regulates responses to Gram-negative bacteria), and Spaetzle/Toll (which generally regulates responses to Gram-positive bacteria and fungi) pathways. Activation of one of these signal transduction pathways may lead to up- or down-regulation of the others depending on the host-parasite system. For example, studies in *Drosophila* have shown that the relationships between immune pathways and effectors can be highly pathogen specific and much still remains to be characterized [25]. Furthermore, canonical pathways derived from *Drosophila* experiments, may not be fully generalizable to other systems.

Honey bees also employ a number of behavioral mechanisms – termed “social immunity” – to reduce the impacts of parasites and diseases (reviewed in [11,26]). Grooming can remove ectoparasites, and hygienic behavior can reduce levels of brood diseases and depress *Varroa* mite populations (mites feed on developing pupae). Workers also coat the interior of their colonies with propolis (derived from plant sap), which has antimicrobial properties and results in lowered levels of immune response genes in individual bees [27]. Furthermore, diseased or otherwise stressed bees undergo accelerated behavioral maturation, moving from in-hive tasks to foraging, which removes them from the brood nest and colony, thereby potentially reducing transmission [28,29]. Finally, studies in termites suggest that healthy nestmates can become “socially vaccinated” by exposure to infected nestmates, thereby improving their resistance to a pathogen [30]. Future investigations may uncover similar defense mechanisms in honey bees.

Honey bees can also use cuticular hydrocarbons as chemical cues to distinguish between healthy and immuno-stimulated adult nestmates, and respond differently to them [31]. Cuticular hydrocarbons are synthesized in oenocytes, which are embedded in the fat body tissue under the epithelia, and deposited on the cuticular surface [32]. Cuticular hydrocarbon patterns can be modified by genotype, physiological state, and environmental context (reviewed in [33,34]), including social status in honey bees [35]. Immunostimulation of honey bee workers with lipopolysaccharides derived from bacterial cell walls caused significant changes in cuticular hydrocarbon profiles in worker bees after four hours, and resulted in altered social interactions [31]. Healthy bees were more aggressive towards their nestmates that were coated with extracts of immunostimulated nestmates than to nestmates that were coated with extracts of healthy nestmates, indicating that these changes in chemical profile could alter worker-worker interactions. Other studies have demonstrated that *Varroa* parasitized pupae and adults have modified cuticular hydrocarbon profiles [36], while infection with a virus can elicit aggressive reactions from healthy nestmates in a colony [37].

Here, we examined the behavioral, chemical and molecular responses in honey bee workers to injection with saline, Sephadex beads, and Gram-negative bacteria (freeze-killed *E. coli* cells), six hours after injection. We determined if these different immune elicitors stimulate unique responses at these three levels; for example, theoretically only *E. coli* injection should activate the Imd pathway, which could lead to distinct chemical profiles and behavioral responses. We also determined if immunostimulation resulted in significantly altered expression of previously annotated honey bee immune genes [9], if there was overlap with other studies of the effects of immunochallenge or parasitization in honey bees and *Drosophila*[38-41], and if there were changes in expression of genes associated with cuticular hydrocarbon biosynthesis pathways.

## Results

### Pilot study on the effects of immune elicitors on locomotion and mortality

Lethality of our treatments was assessed 8 hours after treatment, in a pilot study using bees from Colony 3. After treatment, 1 out of 10 of the control (handled and CO_2_ anesthetized) and 1 of the 10 bead- or bacteria-injected individuals died, respectively. None of the saline-injected workers died.

The behavioral effects of treatments (*N* = 10 for each treatment) were individually assessed on isolated bees placed in circular arenas for 5 minutes as in [31]. Statistical analyses revealed no significant effects of treatments on general activity (total time spent self-grooming: control: 63 ± 23 sec; saline-injected: 56 ± 15 sec; bead-injected: 86 ± 24 sec; bacteria-injected: 83 ± 31 sec; Kruskal-Wallis: H(3)=0.58; p=0.8) or locomotion (number of lines crossed: control: 50 ± 16 sec; saline-injected: 29 ± 8.5 sec; bead-injected: 39 ± 12 sec; bacteria-injected: 43 ± 15.5 sec; Kruskal-Wallis: H(3)=0.507; p = 0.9).

### Effects of immune elicitors on social interactions

To monitor social interactions, we used a nestmate recognition assay in which an individual, 6 hours after treatment, was returned to her cage with her original nestmates and social interactions were monitored. 975 bees from Colony 1, separated in 65 petri dishes, were used for this experiment. This assay has been used regularly in bees and ants to examine nestmate recognition [31,35]. As in the pilot study with isolated individual bees, there was no effect of treatment on locomotion (number of lines crossed: control: 6 ± 4.75; saline-injected: 4 ± 4; bead-injected: 5.5 ± 3; bacteria-injected: 2.5 ± 2.5; Kruskal-Wallis: H(2)=−4.6; p>0.05).

**Figure 1:**
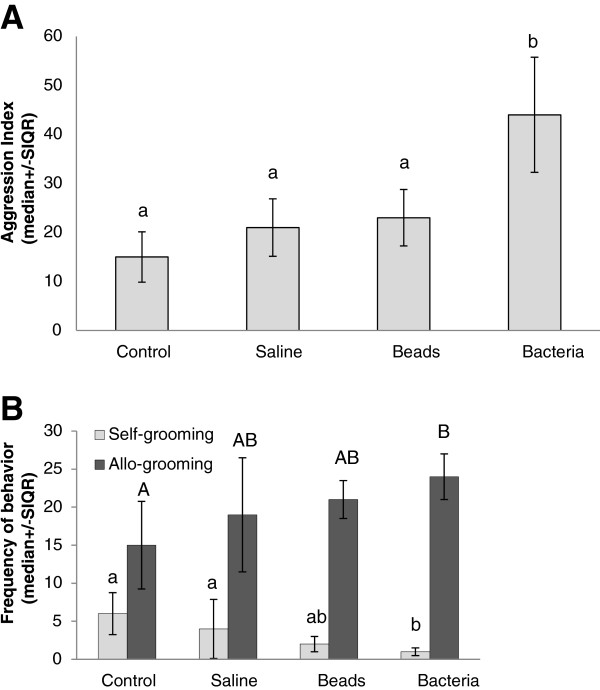
**Effects of immunostimulation on behavioral interactions. A**) Workers are more aggressive towards bacteria-injected nestmates. Behavioral responses of worker bees to treated nestmates were assayed using a caged nestmate recognition assay with bees from Colony 1. Data represents the median ± SIQR aggression index. The number of replicates/treatment is 15 control, 13 saline-, 18 bead-, and 19 bacteria-injected individuals. There were significant differences in behavioural responses across the four treatment groups (H(3)=29.3; p<0.001). Significant differences in post-hoc pairwise comparisons (p<0.05) are denoted by different letters. **B**) Workers engage in more allo-grooming of bacteria-injected nestmates. There were significant differences in allo-grooming of bees in the four treatment groups (H (3, N= 65) =7.97 p =0.046). Significant differences in post-hoc pairwise comparisons (p<0.05) are denoted by different letters.

To assess social interactions, agonistic and non-agonistic contacts were analyzed by calculating a global aggression index (see Table 1). The total aggression index was significantly different among the four groups (Kruskal-Wallis: H(3)=29.3; p<0.001). Pairwise comparisons revealed a significantly higher aggression index toward bacteria-injected workers compared to control, saline-injected and bead-injected workers (Figure 1A).

**Table 1:** Aggression level index of honey bee behavioral interactions

**Abbreviation**	**Behaviour Description**	**Index**
I	Immobility	0
L	Locomotion: walking/running	0
SG	Self-grooming	0
AG	Allo-grooming(antennal contact, grooming or licking)	1
T	Trophallaxis	1
O	Opening mandibles	2
B	Biting	3
F	Biting with gaster flexion	4
S	Stinging	5

Furthermore, unlike the pilot study with isolated bees, the frequency of self-grooming behavior was significantly lower in bacteria-injected workers compared to control and saline-injected workers, while self-grooming by bead-injected workers was intermediate (Kruskal-Wallis: H (3, N= 65) = 15,82 p =0.001 followed by post-hoc pairwise comparisons p<0.01, Figure 1B). However, bacteria-injected workers were the targets of significantly higher allo-grooming behavior than control workers, while saline- and bead-injected workers were intermediate (Kruskal-Wallis: H (3, N= 65) =7,97 p =0.046 followed by pairwise comparisons p<0.05, Figure 1B).

### Effects of immune elicitors on cuticular hydrocarbon profiles

Non-polar compounds were extracted from honey bee workers using pentane, and chemical profiles of cuticular extracts were analyzed using gas chromatography (GC). In our experimental conditions, all the chemical compounds we identified by mass spectrometry (MS) analysis were hydrocarbons.

**Figure 2:**
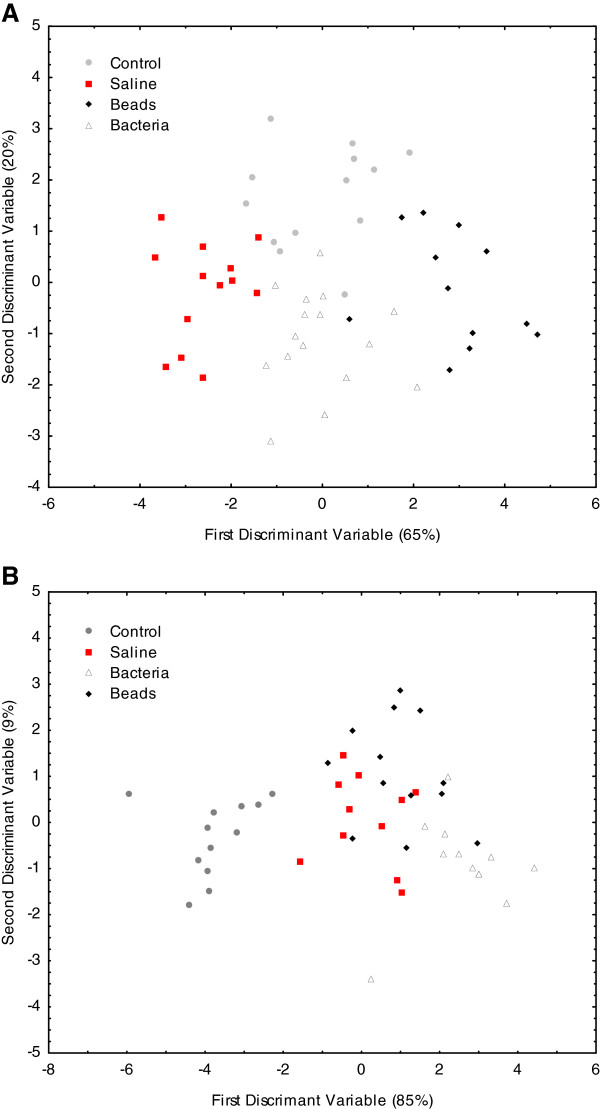
**Effects of immunostimulation on the relative proportions of cuticular hydrocarbons.** Discriminant analysis was performed on the cuticular chemical profiles of control, saline-, bead- and bacteria-injected individuals, based on the relative proportion of each compound. There were significant differences across treatment groups in (**A**) Colony 1, F(_39, 113_)=4.9032; p<0.0001), all Mahalanobis distances, p<0.01, and (**B**) Colony 2, F_(12, 108)_=2.004; p<0.05, all Mahalanobis distances, p<0.05, expect for differences between saline and beads injected individuals, which were p=0.4.

The relative proportions of the cuticular hydrocarbons extracted from all four treatment groups from Colony 1 were significantly different (F_(39, 113)_=4.9032; p<0.0001, Figure 2A and Table 2). Mahalanobis distances between all groups were significantly different (all MD > 8; *P* < 0.01). We obtained similar results for Colony 2 (F_(12, 108)_=2.004; p <0.05, Figure 2B and Table 3). Mahalanobis distances between all groups were significantly different, except between saline- and bead- injected workers (MD<4; p>0.05).

**Table 2:** Effects of immunostimulation on cuticular hydrocarbon profiles of bees in Colony 1

**Substance**	**Control**	**Saline**	**Beads**	**Bacteria**
	**Median ± SIQR**	**Median ± SIQR**	**Median ± SIQR**	**Median ± SIQR**
Alkanes				
Heneicosane	0.51±0.04	0.49±0.08	0.55±0.10	0.43±0.08
Tricosane	2.85±0.24	3.46±0.76	3.70±0.38	3.11±0.60
Tetracosane	0.13±0.02	0.17±0.14	0.22±0.06	0.16±0.08
Pentacosane	4.99±0.37	6.16±6.35	8.89±4.07	5.99±2.91
hexacosane	0.66±0.08	0.83±0.28	0.98±0.19	0.81±0.26
Heptac osane	20.44±2.23	24.00±4.88	29.63±4.40	25.77±8.43
Octacosane	0.78±0.05	0.64±0.10	0.68±0.06	0.73±0.08
Nonacosane	17.65±1.14	13.02±2.77	13.88±3.09	17.14±2.75
Triacontane	0.74±0.07	0.65±0.21	0.47±0.17	0.64±0.15
Hentricontane	16.33±2.37	13.10±4.56	9.92±4.13	15.90±3.63
Tritriacontane	2.16	2.03±0.73	1.35±0.63	2.17±0.73
Alkenes				
Tricosene	0.17±0.03	0.21±0.07	0.19±0.06	0.16±0.04
Pentacosene	0.31±0.05	0.42±0.20	0.37±0.24	0.28±0.12
Heptacosene	0.34±0.13	0.91±0.57	0.72±0.96	0.51±0.35
Nonacosene	1.08±0.28	1.41±0.22	1.23±0.20	1.16±0.13
Hentriacontene Isomere1	3.59±0.25	4.13±0.77	3.80±0.32	3.32±0.61
Hentriacontene Isomere2	5.13±0.30	5.32±1.11	4.81±0.54	4.52±0.63
Tritriacontene	12.99±1.19	13.66±2.58	11.53±1.27	11.11±2.13
Alkynes				
Hentriacontyne	0.12±0.10	0.37±0.06	0.00±0.00	0.18±0.05
Tritriacontyne	1.15±0.28	1.61±0.32	1.34±0.32	1.14±0.21
Methylalkanes				
11.13- Methylpentacosane	0.20±0.04	0.20±0.07	0.27±0.07	0.17±0.04
9.11.13- Methylheptacosane	1.15±0.19	1.40±0.25	1.70±0.40	1.20±0.24
11.13.15- Methylnonacosane	1.06±0.18	1.34±0.25	1.67±0.35	1.11±0.22
13.15- Methylhentriacontane	0.62±0.11	0.85±0.15	1.01±0.21	0.74±0.14

Data for this figure were obtained from gas chromatography analysis of cuticular washes of worker bees from Colony 1. These data represent the relative proportions of each compound found within each of the four treatment groups.

**Table 3:** Effects of immunostimulation on cuticular hydrocarbon profiles of bees in Colony 2

**Substance**	**Control**	**Saline**	**Beads**	**Bacteria**
	**Median ± SIQR**	**Median ± SIQR**	**Median ± SIQR**	**Median ± SIQR**
Alkanes				
Heneicosane	0.84±0.10	0.98±0.10	0.82±0.06	0.87±0.13
Tricosane	7.55±3.26	8.25±1.26	8.65±1.84	10.28±2.53
Tetracosane	0.45±0.13	0.46±0.09	0.46±0.06	0.54±0.06
Pentacosane	19.70±4.82	17.89±4.25	17.94±1.90	19.85±2.57
hexacosane	1.12±0.11	1.23±0.11	1.20±0.04	1.09±0.14
heptacosane	27.03±3.75	29.08±2.38	28.24±1.58	27.46±3.37
Octacosane	0.52±0.08	0.57±0.07	0.63±0.06	0.54±0.04
Nonacosane	7.82±2.12	8.52±1.58	9.11±1.18	7.71±1.52
Hentricontane	6.09±1.29	6.81±1.34	6.25±1.67	6.64±1.80
Tritriacontane	0.89±0.23	1.25±0.27	0.93±0.37	1.16±0.36
Alkenes				
Tricosene	0.93±0.43	0.79±0.20	0.76±0.27	1.07±0.35
Pentacosene	2.30±0.81	2.28±0.46	2.24±0.40	2.53±0.47
Heptacosene	0.49±0.07	0.51±0.12	0.55±0.17	0.50±0.13
Nonacosene	1.70±0.26	1.94±0.20	1.81±0.33	1.55±0.19
Hentriacontene Isomere 2	2.89±0.78	3.51±0.86	2.64±0.83	2.68±0.42
Tritriacontene	7.12±1.27	7.65±1.43	5.96±1.04	6.41±1.34
Alkynes				
Heptacosyne	1.93±0.23	2.18±0.41	2.14±0.26	2.08±0.27
Tritriacontyne	1.04±0.23	1.08±0.19	1.00±0.22	0.80±0.08
Methylalkanes				
9.11.13-Methylheptacosane	1.53±0.25	1.59±0.18	1.48±0.18	1.30±0.22
11.13.15-Methylnonacosane	1.61±0.30	1.70±0.17	1.56±0.12	1.42±0.25
13.15-Methylhentriacontane	0.92±0.19	0.97±0.11	0.91±0.07	0.82±0.15

Data for this figure were obtained from gas chromatography analysis of cuticular washes of worker bees from Colony 2. These data represent the relative proportions of each compound found within each of the four treatment groups.

**Figure 3:**
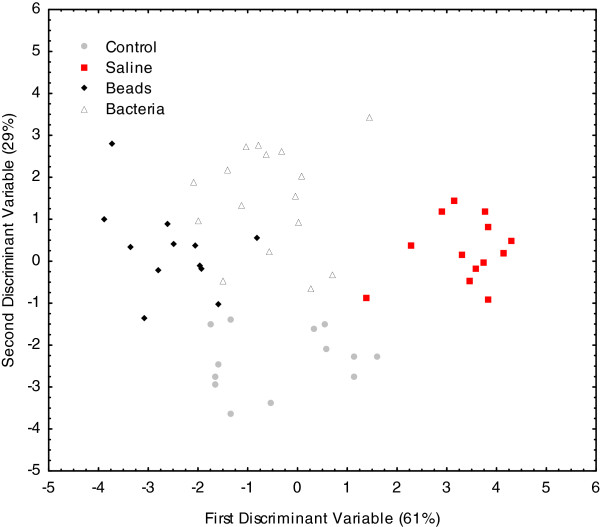
**Effects of immunostimulation on the absolute quantities of cuticular hydrocarbons.** Discriminant analysis was used to determine if there were significant differences in the cuticular hydrocarbon profiles of control, saline-, bead- and bacteria-injected individuals, based on absolute quantity of each compound. There were significant differences across all treatments F(51, 102)=4.32; p<0.0001.

Discriminant analyses based on the absolute quantities of cuticular chemical compounds also revealed significant differences between the four treatment groups (F_(51, 102)_=4.32; p<0.0001 for Colony 1, Figure 3, and F_(45, 89)_=3.03; p<0.0001 for Colony 2, data not shown). There did not appear to be a significant and consistent effect of treatment on the relative proportions or quantities of any of the major chemical classes across the two colonies. The total quantity of branched alkanes decreased in both colonies in the bacteria-injected workers, but this difference was not significant (data not shown).

### Global gene expression responses to immunostimulation

**Figure 4:**
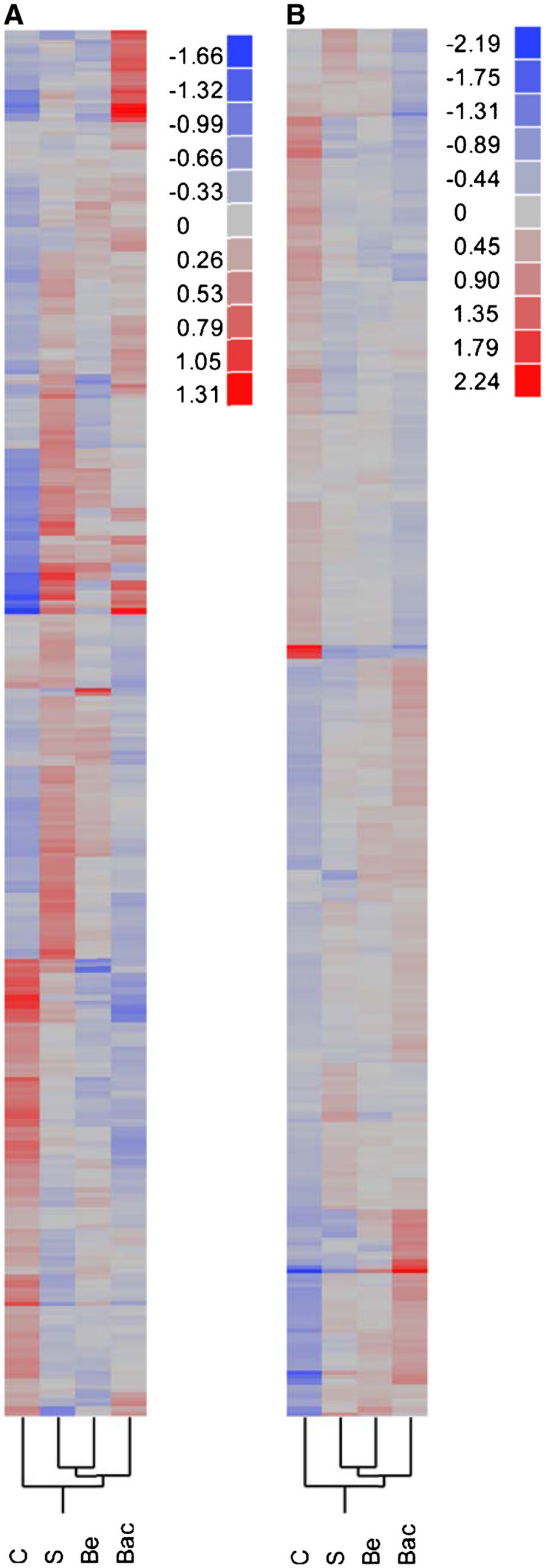
**Hierarchical clustering of significantly regulated genes.** We performed hierarchical clustering analysis on the 302 significantly, differentially expressed transcripts (FDR<0.01) similarly regulated in Colony 1 (**A**) and Colony 2 (**B**). Both colonies demonstrated the same overall grouping for experimental treatments: saline (S) and bead (B) injected bees formed a sister group and were closer in transcript expression to bacteria (Bac) injected bees than controls (**C**). Control and bacteria-injected bees had the most disparate transcript expression. Colours denote differences in log2 expression relative to the mean expression across the four treatment groups, according to the scale shown.

Microarrays were used to monitor global gene expression patterns in the eviscerated abdomens (containing epithelial tissue, fat bodies, and oenocytes) of bees from the four treatment groups. We examined all transcript expression levels across all pairs of treatment groups for significant differences (control x saline, control x bead, control x bacteria, saline x bead, saline x bacteria, bead x bacteria) and found that 670 unique transcripts were significantly regulated among treatment groups in Colony 1, and 1610 unique transcripts were significantly regulated among treatment groups in Colony 2 (FDR <0.01, see Tables S1 and S2 (Additional file 1) for a list of transcripts). Thus, expression levels for these transcripts were significantly different between at least two of the treatment groups. Of these, 302 transcripts were significantly regulated in both colonies (see Additional file 1: Table S3a and S3b). This overlap in transcript expression was greater than expected by chance (Fisher’s Exact Test; p<0.001). The higher numbers of transcripts in Colony 2 may be due to the lower number of biological replicates (4 vs 6 replicates) or the effect of genetic background, which can strongly affect responses to immunostimulation [42,43]. Despite some differences in relative expression patterns for individual transcripts across colonies, hierarchical clustering of the 302 common transcripts revealed the same overall clustering of treatment groups, (Figure 4). Treated groups clustered separately from control groups, and saline- and bead-injected groups clustered separately from the bacteria- injected group. Relative-fold expression values for these transcripts across all of the treatment groups can be found in (Additional file 1: Table S3a), and the associated p-values for the pairwise comparisons between treatments can be found in (Additional file 1: Table S3b).

### Effects of individual immune elicitors on gene expression

**Figure 5:**
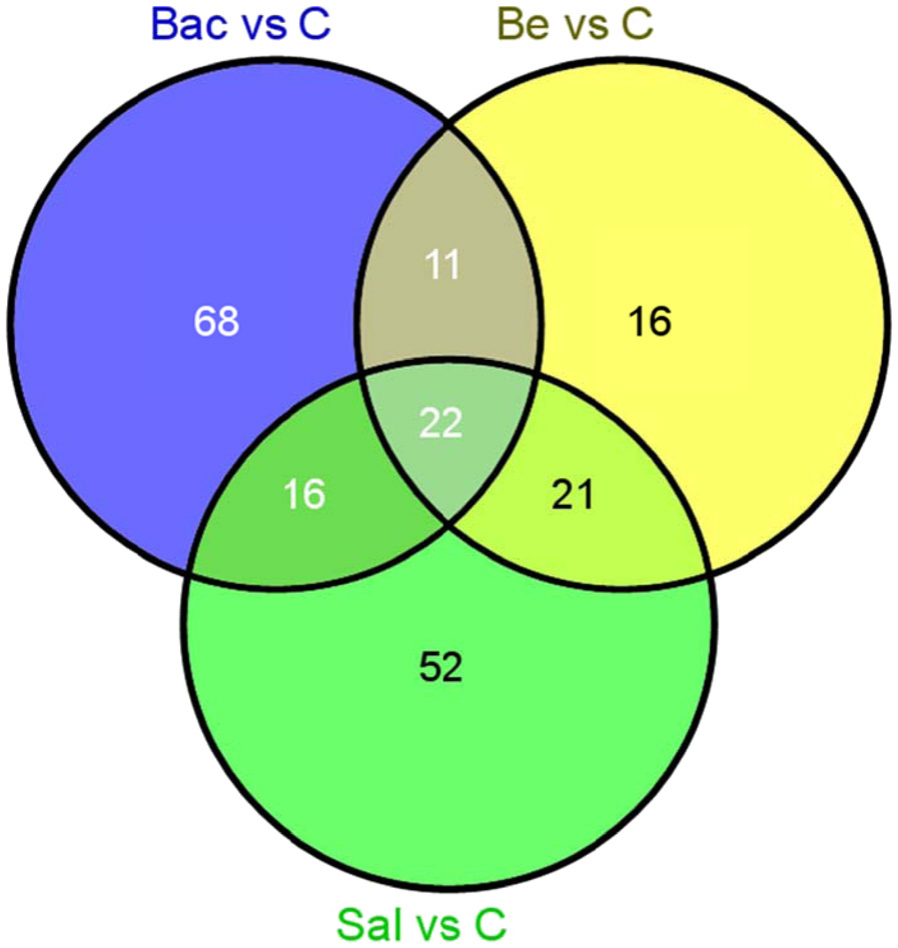
**Effects of specific immunostimulants on gene expression.** Pairwise comparisons identified sets of transcripts differentially regulated between saline-, bead-, bacteria-injected bees and control bees in both colonies. A Venn diagram demonstrates that there is considerable overlap in the effects of each treatment, but also treatment-specific effects on gene expression. The numbers represent the number of genes in each category.

Pairwise comparisons identified sets of transcripts differentially regulated between saline-, bead-, bacteria-injected bees and the control bees (FDR < 0.01, Table 4). Overlap between colonies for each treatment-control comparison was significantly greater than expected by chance (Table 4, see Additional file 1: Table S4 for a listing of these transcripts). 111, 70, and 117 transcripts, respectively, were significantly regulated in these pairwise comparisons in both colonies, though these transcripts did not necessarily show the same directional patterns of expression between colonies. While each treatment resulted in significant expression changes in a unique set of transcripts relative to controls, there was considerable overlap across treatment groups (Figure 5). Indeed, 22 transcripts were significantly regulated by all three treatments (Figure 5; see Additional file 1: Table S5 for a listing of these transcripts).

**Table 4:** Analysis of overlap among treatment groups

**Treatment group**	**# of significantly, differentially regulated transcripts**			**p-value (Fisher's Exact Test)**
	**Colony 1**	**Colony 2**	**In both**	
Saline x Control	340	555	111	p < 0.001
Bead x Control	213	398	70	p < 0.001
Bacteria x Control	278	1198	117	p < 0.001
Total	*557	*1453	*206	

Fisher exact tests found more overlap than expected (p<0.001) for significantly, differentially expressed transcripts (FDR<0.01) in the saline-, bead- and bacteria-injected vs control groups between the two colonies.

*duplicate transcripts between groups removed.

### Functional analysis of regulated genes

Gene ontology analysis of the significantly regulated 302 transcripts found in both colonies (207 of which had unique *Drosophila* orthologs with Flybase annotations and thus were used in the analysis) revealed an overrepresentation of genes involved in immune response, spermatogenesis, wing disc dorsal/ventral pattern formation, tissue development, post-transcriptional regulation of gene expression and protein polymerization (p<0.05, see Additional file 1: Table S6 for a listing of the genes in these GO categories). However, none of these categories survived the Benjamini correction. Immune genes corresponding to the major immune pathways were significantly regulated (*defensin-1*, *relish*, *domeless*, *cactus*, *melanization protease 1*, *death related ced-3/DREDD, PGRP-SC2*, *kayak, spirit* among others). Pale (*ple*), a tyrosine hydroxylase involved in melanization and wound repair, was also significantly up-regulated by bacteria, bead and saline injection in Colony 1 and bacteria and bead injection in Colony 2 [44]. Other genes included those involved in cell growth and proliferation (*insulin-like receptor*), cytoskeleton structure (*basigin, chickadee, twinstar, annexin ix,* and isoforms of *tubulin*), extracellular matrix components (*pericardin* and *laminin A*), Notch signaling (*apterous, pebbled, groucho*), phagocytosis (*draper*), and *cabut*, which encodes a transcription factor that is regulated by the JNK cascade [45].

Of the 22 transcripts (corresponding to 17 unique Flybase genes) significantly regulated by injection with saline, beads, and bacteria relative to controls, two categories were significantly overrepresented: organ morphogenesis, p<0.005, and developmental process, p<0.001 (see Additional file 1: Table S7 for a listing of genes in these GO categories). Again, neither of these categories survived the Benjamini correction. Several of the previously discussed genes (*domeless*, *insulin-like receptor*, *basigin*, *twinstar*, and *groucho*) are part of this group, as well a serine protease immune response integrator (*spirit*) which functions in Toll pathway activation [46]. Notably, two genes involved in lipid metabolism were also found in this group, which is of particular interest since fatty acids are the precursors of cuticular hydrocarbon synthesis in insects (reviewed in [47]). *Bubblegum* (*bgm*) encodes a very long chain fatty acid CoA ligase, which plays a role in fatty acid metabolism, and was down-regulated in the treatment groups [48]. *Lipid-storage droplet 2* (*Lsd-2*) is involved in lipid storage and accumulation, and was up-regulated in the treatment groups [49]. *Lipid storage droplet 1* (*Lsd-1*), a component of lipid droplets in fat bodies [50], was found to be significantly down-regulated in both colonies, but not by all treatments.

GO analysis of the 68 transcripts whose expression was specifically altered in bacteria-injected workers (corresponding to 50 unique Flybase genes) yielded only one significantly overrepresented cluster (immune response; p=0.001). These genes included *cactus*, *kayak*, *draper*, *defensin-1*, *relish*, *PGRP-SC2*. Other regulated genes included those that may be involved in metabolism, such as *Gr28b*, a gustatory receptor that seems to mediate diet-related changes in immune response [51], *sorbital dehydrogenase-2* (*Sodh-2*), an alcohol dehydrogenase of sugars [52], and *trehalose-6-phospate synthetase 1* (*Tps1*) which may play a role in protection from hypoxia and anoxic injury [53]. See Additional file 1: Tables S8 and S9 for a listing of the bacteria-regulated genes and GO categories.

### Comparisons of gene expression patterns with previous studies

We examined overlap in gene expression patterns between our study and previous studies in honey bees and *Drosophila*. It is important to note that there were large differences in study design (including pathogen challenge, timecourse and tissue) as well as gene expression platform and statistical analyses, which makes direct comparisons challenging. However we sought to identify common regulated genes that could indicate conserved immune response mechanisms across different host organisms and pathogen challenges.

Fourteen genes were found in common between the significantly regulated 302 transcripts (239 of which were annotated honey bee genes with GB identifiers) and the canonical immune response genes annotated from the honey bee genome by Evans and colleagues [9] (see Additional file 1: Table S10 for a listing of these genes). This overlap was significantly greater than expected by chance (Fisher’s Exact Test, p<0.0001; Table 5). However, clearly the majority of significantly regulated genes are not members of these canonical immune response pathways.

**Table 5:** Comparisons of significantly regulated genes with previous studies

**Gene list**	**# of significant transcripts**	**# of transcripts in common between studies**	**p-value (Fisher's Exact Test)**
Evans et al. ( [2006])	166	14	p < 0.0001
Navajas et al. ( [2008])	22	2	p > 0.09
Alaux et al. ( [2011])	3704	117	p < 0.0001

The 302 genes that were significantly regulated by immunostimulation in both colonies in our study were matched to 239 GB numbers and compared with the immune genes identified during annotation of the genome (Evans et al., [2006]), and genes significantly regulated by *Varroa* parasitization of honey bees (Navajas et al., [2008] and Alaux et al., [2011]). A Fischer’s Exact Test was used to determine if this overlap was significant, assuming that all genes present in the background lists for the arrays were in common between studies. In order to ascertain overlap, only genes in previous studies that were present on our array platform were included.

Navajas et al. [40] performed a microarray analysis of control and *Varroa* mite-parasitized honey bee pupae from two strains (resistant and sensitive to *Varroa* mite infestation). Expression levels of 32 genes (with 22 matching unique GB identifiers) were significantly altered by *Varroa* parasitization. There was no significant overlap with the 302 transcripts (matched to 239 GB identifiers) in the current study (Fisher’s Exact Test, p>0.09; Table 5). However, two genes, including *ple*, were regulated in both studies, though directionality was not necessarily conserved between studies.

In a separate study, Alaux and colleagues examined the effects of *Varroa* parasitization and nutrition on honey bee worker gene expression using an RNA-seq approach [38]. We compared our 302 regulated transcripts (matched to 239 GB identifiers) with the list of genes that were significantly, differentially regulated by mite parasitization in pollen-fed bees and found 117 overlapping genes, including genes involved in immune and metabolic processes. Though directionality was not necessarily conserved between studies, the overlap was greater than expected by chance (Fisher’s Exact Test, p<0.0001; Table 5, see Additional file 1: Table S11 for a listing of overlapping genes). *Bgm*, *Lsd-2*, and *domeless* were among the genes significantly regulated in both studies. A GO analysis of the overlapping transcripts (corresponding to 102 unique Flybase genes) found one significantly overrepresented category which did not survive Benjamini correction (metabolic process, p<0.04).

Finally, we compared the 302 significantly regulated transcripts in our study (matched to 207 unique Flybase identifiers) with significantly regulated genes in two separate studies examining immune response in fruit flies. De Gregario and colleagues [39] identified 400 significantly regulated genes after septic injury of male *Drosophila* with bacteria contaminated needles or feeding with fungal spores (corresponding to 497 unique Flybase genes). Roxstrom-Linquist and colleagues [41] identified approximately 390 upregulated genes (corresponding to 464 unique Flybase genes) in *Drosophila melanogaster* orally infected with protozoa, viruses, bacteria or fungi. Eight genes were found in all three studies (Additional file 1: Table S12), including a number of genes with immune related functions: *ple*, *cactus, defensin-1, relish* and *PGRP-SC2.* Serpin 28D (CG7219*)* was also regulated in all three studies, and is involved in melanization [54].

### Quantitative real-time PCR validation of expression of candidate genes

**Figure 6:**
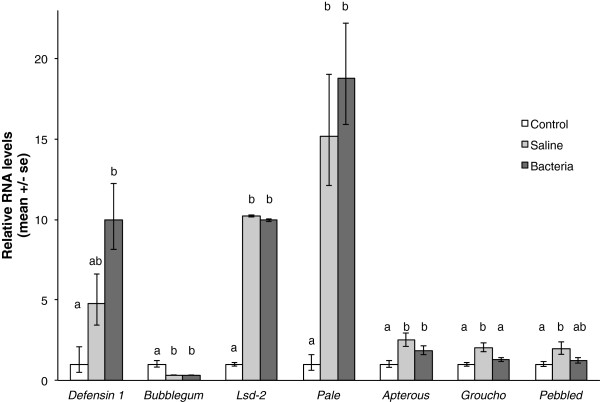
**Quantitative real-time PCR validation of expression patterns of candidate genes.** Expression levels of seven candidate genes (relative to actin) were analysed in a third biological replicate using quantitative real-time PCR. Mean expression levels for each treatment group are normalized to expression in the control treatment group, for graphical representation. Significant differences in expression levels across the three treatment groups were determined using an ANOVA with treatment as a variable, followed by post-hoc Tukey HSD pairwise comparisons. Different letters denote significant differences in expression (p<0.05). Nine, eight, and ten individual bees were used for the control, saline-injected, and bacteria-injected treatment groups, respectively.

We examined expression levels of several candidate genes identified in the microarray study in a third biological replicate (Figure 6). Three treatment groups were used: control, saline-injected, and bacteria-injected worker bees. As in the microarray study, *defensin 1* levels increased with treatment, and were significantly higher in bacteria-injected bees relative to controls, and intermediate in saline-injected bees (F(2,24)=6.49, p=0.0056, see Figure 6 for results of Tukey HSD post-hoc pairwise comparisons and Additional file 1: Table S3a for expression levels obtained from the microarray analysis). As in the microarray study, levels of *bubblegum* decreased significantly with treatment (F(2,24)=32.56, p<0.0001), while levels of *Lsd-2* (F(2,24)=392.1, p<0.0001) and *pale* increased (F(2,24)= 27.98, p<0.0001). Similar to the results of the microarray analysis, the effects of treatment on expression of three genes involved in Notch signaling were less dramatic, but nonetheless expression of *apterous* (F(2,24)=6.76, p=0.0047), *groucho* (F(2,24)=8.44, p=0.0017) and *pebbled* (F(2,24)=4.41, p=0.023) were significantly affected by treatment. Expression levels were higher in saline-injected bees than controls for all three genes, and higher in bacteria-injected bees relative to control bees for *apterous*.

## Discussion

We have demonstrated that immunostimulation of adult honey bee workers results in altered social interactions only in the case of bacteria-injected bees. However, all the treatment groups have significantly different cuticular hydrocarbon chemical profiles, and all treatments caused large scale changes in gene expression patterns in abdominal fat body and epithelial tissue, which includes oenocytes. It is important to note that these dramatic changes occurred in a relatively short timeframe, only six hours after immunostimulation. Importantly, this study demonstrates that expression of a large number of genes, not just those canonically associated with immune response pathways, are modulated by immunostimulation, as has been demonstrated by several other studies of genome-wide responses to immune challenges (for example, [38-41]). However, comparisons with studies in *Drosophila* found limited overlap of gene expression patterns, and the handful of commonly regulated genes were key members of canonical immune response pathways.

It is somewhat perplexing that only bacteria-injected bees were subjected to significantly increased allo-grooming and aggression. All treatments caused changes in chemical profiles, and the changes did not appear to be substantially larger in bacteria-injected bees, though there was a trend for lower total quantity of branched alkanes in this group. However, previous studies have suggested that alkenes, rather than alkanes, play a significant role in nestmate recognition [55]. Based on the hierarchical clustering analysis, the gene expression patterns of bacteria-injected bees were clearly distinct from saline- and bead-injected bees. However, overall it did not appear that bacterial injection altered expression of an entirely unique set of genes (out of 302 transcripts, expression of 68 transcripts were significantly regulated only by bacterial injection, relative to controls, and expression of many of these genes changed non-significantly in the other treatments as well). Thus, perhaps bacteria injection did not cause change in expression of a large, unique subset genes, but rather caused more substantial changes in the magnitude of expression levels of some genes, as indicated by hierarchical clustering. Alternatively, it may be that expression of a few key genes and/or chemical profile components in bacteria-treated bees provoked behavioral changes in nestmates.

It remains to be determined if infection with honey bee specific parasites and pathogens causes similar changes in gene expression and behavioral responses, though there is some indication that infection with *Nosema* microsporidia does stimulate expression changes in a significantly overlapping set of genes (Holt, Aronstein and Grozinger, unpublished data). It also remains to be determined if these behavioral changes are adaptive: it is possible that bees have evolved to “match” the strength of the signal to the virulence of the pathogen, and thus infection with bacteria would elicit a greater change in the behavioral responses of nestmates than simple cuticular wounding. However, colony-level assays would need to be performed in order to determine if these changes in social interactions actually result in differences in the spread of pathogens through the colony and their impacts on infected individuals. For example, the increased grooming behavior we observed towards bacteria-injected individuals could facilitate the spread of pathogens through the colony (as observed in carpenter ants, [56]), slow the spread by causing isolation or removal of infected individuals [56,57] or reduce the impact on the infected individual [58,59].

Our studies also demonstrate that immunostimulation elicits complex gene expression changes in the epithelial tissues in honey bee workers. Immunostimulation resulted in significant expression changes of 302 common transcripts in worker bees from the two colonies examined. Only 14 of these genes corresponded to previously annotated immune genes identified from the honey bee genome [9]. Several other biological processes were modified, including cell growth and proliferation, cytoskelatal structure, metabolism and components of the Notch signaling pathway. Cell growth, proliferation, and migration, particularly involving actin-mediated cytoskeletal changes, are required for repairing epithelial wounds [24]. The Notch signaling pathway has not yet been linked to immune response or wound repair, but wound repair uses many of the same developmental pathways that function during dorsal closure in *Drosophila* development, which does involve Notch signaling [60]. Alternatively, since the insect fat body is involved in regulating many key processes, including metabolism (which was commonly regulated in our study and by *Varroa* parasitization [38]), these changes may reflect general physiological changes after immunostimulation or stress [61]. Expression changes in three genes of the Notch signaling pathway (*apterous*, *groucho*, and *pebbled*) were confirmed using quantitative real-time PCR. Interestingly, expression was significantly higher in saline-injected bees relative to controls for all three genes, but only expression of apterous was affected in bacteria-injected bees, suggesting that changes in Notch signaling may be modulated temporally or by other signaling pathways.

We found significant expression changes of a number of key immune response genes (for a review of the function of these genes, see [10,23,24]). The JAK/STAT pathway is regulated by the Domeless receptor; *domeless* expression was significantly regulated by immune stimulation in both genotypes of bees in our study. Activation of the IMD pathway requires cleavage of Relish by the caspase DREDD; both *Dredd* and *Relish* were significantly regulated in our study. We also observed significant regulation of *PGRP-SC2* which suppresses activation the IMD pathway, with bacteria-injected bees showing the highest levels of PGRP-SC2 expression (data not shown). In fruit flies, PGRP-SC1 and PGR-SC2 may function in preventing over-activation of the IMD pathway [62]. The IMD pathway triggers the JNK pathway, which activates the transcriptional regulator AP-1 (which contains Kayak/D-fos), and AP-1 in turn negatively regulates Relish-dependent transcription. We found significant regulation of *kayak* in our study. Interestingly, we also found significant changes in expression of *cabut*, which is regulated by the JNK pathway but has not yet been linked to immune function [45]. The Toll pathway operates through transduction factors including *spirit,* which acts extracellularly and upstream of Spaetzle [46] and the NF-κB protein Dorsal (which is negatively regulated by *cactus*). We found significant regulation of *spirit* and *cactus* in our study. *Pale*, which plays an important role in melanization and wound repair, and *draper*, which functions in phagocytosis, were also significantly regulated in our study. As a Gram-negative bacteria, it would be expected that *E. coli* would primarily stimulate activation of the IMD pathway. However, we observed changes in gene expression of members of the Toll pathway, including *cactus*, *spirit* and *defensin-1,* which exhibits Gram-positive antimicrobial activity and is not regulated by the IMD pathway [63]. Thus, there is likely considerable cross-talk between the pathways.

Our studies also identified several genes which may play a role in altering cuticular hydrocarbon patterns. Cuticular hydrocarbons are synthesized primarily in the oenocytes (reviewed in [47]), which are embedded in the fat body of adult honey bees [64]. Cuticular hydrocarbon biosynthesis involves activation of fatty acids by an acyl-CoA synthetase, chain elongations of fatty-acyl-CoAs to produce very long chain fatty acids, and subsequent conversion to a hydrocarbon, likely by a p450 enzyme [47]. Fatty acids are stored in lipid droplets in the adipoctye cells of the insect fat body [61]. Fatty acids can be released from droplets in the adipocytes and accumulate in the oenocytes; this occurs under starvation conditions in particular [65]. This process is mediated in part by lipid storage droplet-2 (*Lsd-2*): increased *Lsd-2* expression in the fat bodies decreases lipid movement to the oenocytes. We found increased expression of *Lsd-2* in immunostimulated bees (see Figure 6), suggesting reduced movement of lipids to oenocytes, and perhaps reduced levels of cuticular hydrocarbons. We did observe a decrease in the total relative quantity of all branched alkanes in bacteria-injected workers in both colonies but this difference was not significant.

*Bubblegum* (*bgm*) activates long chain fatty acids to form acyl-CoAs (reviewed in [66]), a key step in cuticular hydrocarbon biosynthesis. *Bgm* was originally described as a *Drosophila* mutant that resulted in elevated levels of very long chain fatty acids and neurodegeneration [48]. Bgm homologs have been identified in numerous species, including humans and mice, and have been demonstrated to activate long chain (C16) and very long chain (C24) fatty acids [67]. In our study, *bgm* expression was significantly decreased relative to controls (see Figure 6).

Despite large differences in study designs and analysis methods, we found some overlap in gene expression with previous studies examining the effects of *Varroa* mite parasitization on honey bees [38,40]. *Varroa*-responsive genes were significantly associated with basic cellular processes, including cell organization, biogenesis and metabolism. We also found significant changes in functional categories associated with basic cellular processes, such as cell growth, proliferation and cytoskeletal structure. *Varroa* parasitization also caused changes in expression of *pale*. As discussed above, this may represent cellular mechanisms for wound-healing. Expression of potential hydrocarbon synthesis genes, namely *bgm* and *Lsd-2*, were also regulated by *Varroa* parasitization. Indeed, *Varroa* parasitized pupae and adults have modified cuticular hydrocarbon profiles [36]. These differences are likely responsible for stimulating hygienic behavior, in which diseased larvae are removed by adult worker bees, a key component of *Varroa* resistance [11].

Comparison with two previous studies [39,41] examining the effects of immunostimulation on *Drosophila* global gene expression patterns revealed conserved changes in expression of key immune genes in *Drosophila* and honey bees (including *relish*, *cactus*, *defensin-1*, *spirit, PGRP-SC2*, and *pale)*, but otherwise limited overlap in the significantly regulated genes. The lack of similarity could represent species-specific immune responses or simply technical differences – for example, Roxstrom-Lindquist [41] orally infected young male flies with bacteria, fungi and microsporidia, and measured whole-body gene expression changes in only two replicates using Affymetrix microarrays.

## Conclusions

Our results suggest that immunostimulation of honey bee workers causes significant changes in gene expression patterns, cuticular hydrocarbon chemical profiles, and, in the case of bacteria-injection, social behavior. While bacteria-injection did not cause expression changes in an entirely unique set of genes or production of unique cuticular hydrocarbons compared to other treatment groups, it does appear that the magnitude of the expression and production changes was greater, and this may have resulted in the altered behavioral responses. As demonstrated by other studies examining genome-wide expression changes associated with immune challenges [38-41], we found that even a short-term immune challenge can result in dramatic changes in gene expression that encompass far more genes than those represented by canonical immune response pathways. For example, we found several genes associated with the Notch signaling pathway, which suggests this pathway may also play a role in mediating immune responses. Furthermore, our study has highlighted potentially new candidate genes for regulating cuticular hydrocarbon synthesis in insects. These chemicals serve many functions in insects, including operating as sex and caste-specific pheromones, and most research on their biosynthesis has focused on desaturase enzymes and p450s [34]. These studies lay the groundwork for future work examining the molecular pathways that mediate immune responses to acute stimulators and chronic infections in bees and other insects, and has paved the way for research into the genes that regulate social immunity.

## Methods

### Honey bee stocks

Honey bee colonies were maintained according to standard practices at the Lake Wheeler Honey Bee Research Facility at North Carolina State University in 2008. Workers for these studies were obtained from three source colonies, headed by queens each instrumentally inseminated with semen from a single, different, male (Glenn Apiaries, Fallbrook, CA). The specific source colonies used for the different experiments were Colonies 1, 2 and 3, and are listed in the experimental details below. Since honey bees are haplodiploid, the coefficient of relatedness among the workers in each colony was 0.75, thereby reducing variation in gene expression responses, chemical profiles, and presumably behavioral responses among the nestmates. Honeycomb frames of late-stage pupae were removed from the colonies and placed in incubators overnight at 33°C, 50% relative humidity (RH).

### Honey bee rearing

Newly emerged bees (<12 hours old) were brushed from the frames and placed into modified 10 cm Petri dishes in groups of 15. Dishes were maintained under red light in a temperature and humidity-controlled environmental room (33°C, 50% RH, Phytotron Facility, NCSU). Bees were fed 50% sucrose/water solutions and 45% honey/45% pollen/10% water paste ad libitum. Food was replaced every two days. Bees were also exposed to 0.1 queen bee equivalents of queen mandibular pheromone (QMP) (Pherotech, Vancouver, Canada). Every day, 10 μl of QMP (0.01 queen equivalents/μL in an isopropanol/1% water solution) was placed on a microscope slide and allowed to evaporate before being placed in the cage. This amount of QMP mimics a live queen in assays of worker behavior and physiology [68,69] and thus should help simulate normal rearing conditions.

### Experimental treatment

When the bees were 10 days old, five individual bees were removed from each cage (leaving 10 in the cage). Three of these were then subjected to one of four treatments. The first group of bees was handled and anesthetized with CO_2_ for 1 minute (control treatment). A second group of bees was anesthetized and injected with 8 μl of sterile bee saline (130 mM NaCl, 6 mM KCl, 4 mM MgCl_2_, 5 mM CaCl_2_, 160 mM sucrose, 25 mM glucose, 10 mM 4-(2-hydroxyethyl)-1-piperazineethanesulfonic acid in distilled water, pH 6.7, 500 mOsmol, as in [31]). A third group of bees was anesthetized and injected with 8 μl of a CM-25 Sephadex beads (Sigma-Aldrich, Steinheim, Germany) solution (0.01 g of beads mixed with 500 μl sterile bee saline and vortexed, resulting in approximately 110 beads/8 μl). The fourth group of bees was anesthetized and injected with 8 μL of an *E. coli* (JM101, Sigma, St Louis, MO) solution suspended in sterile bee saline (3.8*10^5^ cells/bee, following a protocol modified from [70]). The bacteria were grown in LB media (Sigma, St Louis, MO), collected and resuspended in the sterile bee saline at the appropriate concentration (4.75*10^4^ cells/μL), and then stored at −80°C until it was thawed for the injections. Injections were performed into the abdominal cavity through tergites with a nano-injector equipped with a glass needle (Schley Compact Model II Instrument; Honey Bee Insemination Services, Davis, CA, US), using a binocular microscope (Leica MZ6 stereomicroscope, Leica Microsystems, Buffalo Grove, IL). Treated bees were marked with a dot of Testor’s paint on their thorax and maintained individually in 10x10x7 cm^3^ Plexigas cages in an incubator under red light at 33°C, 50%RH for 6 hours, with 50% sucrose. After 6 hours, two treated bees per cage were collected onto dry ice and stored at −80°C for microarray or chemical analysis, while the third was reintroduced (individually) to her original Petri dish cages for behavioral analysis.

Behavioral analysis was performed on bees from Colony 1 (15 control, 13 saline-, 18 bead-, and 19 bacteria-injected individuals), while chemical analysis was performed on bees from Colony 1 (13 control, 13 saline-, 12 bead- and 16 bacteria-injected individuals) and Colony 2 (12 control, 11 saline-, 13 bead- and 12 bacteria-injected individuals). Microarray analysis was performed on 6 individuals/treatment in Colony 1, and 4 individuals/treatment in Colony 2. Colony 3 was used for pilot studies of the deleterious effects of the treatments (see below).

### Behavioral assessment of the deleterious effects of treatment

#### Assessment of grooming behavior and locomotion

To determine whether the treatments induced any deleterious effects, we conducted a preliminary study in 10-day-old honey bees (*N* = 10 per treatment, from Colony 3). Immediately following the injection, individual workers were placed into the Petri dish cages, where they were later observed. Grooming behavior and locomotion were assessed after four hours. To assess locomotor activity, a cross was drawn under the circular arena and the number of lines crossed during 5 minutes was used as a locomotion index. All studies were conducted blind to the treatment group. Mortality was assessed 8 hours after treatment and only two workers died.

#### Assessment of social interactions

Six hours after being exposed to one of the four treatments, individual workers were reintroduced to their original Petri dish cage. Assays were conducted blind to the treatment group. Each cage was used only once. Beginning 30 seconds after the reintroduction, the number and type of social behaviors of the non-treated bees toward the treated bee were recorded through a scanning method, every 10 seconds for 5 minutes. Aggression levels were defined using standard practices, as described in Table 1[31,35]. For each treated bee, the aggression score was calculated by multiplying the number of non-treated bees observed performing a particular behavior during the observation period by the aggression level index (Table 1) for that behavior, and summing across all interactions observed.

### Hydrocarbon extraction and analysis

Cuticular hydrocarbons were extracted by submerging each frozen bee individually into 1 ml n-pentane with 10 μg of hexadecane (Sigma, St Louis, MO, U.S.A.) as an internal standard, and agitating gently for 10 min. Whole bees were used for all samples. The solvent was reduced to dryness with a gentle stream of N_2_, and the sample resuspended in 100 μl. Hydrocarbons were quantified by gas chromatography and identified by gas-chromatography–mass spectrometry (GC–MS). We analyzed 2 μl of solution on a HP5890II GC (Agilent, Palo Alto, CA, U.S.A.) equipped with a flame-ionization detector and interfaced with a HP ChemStation (Rev. A.09.03). Splitless injection was made into a 30 m x 0.32 mm x1 μm HP-5 capillary column operated at 150°C for 2 min, increased at 15°C/min to 250°C then at 3°C/min to 300°C and held at this temperature for 15 min. The injector and detector were held at 300°C and 310°C, respectively. Compound identification was achieved by splitless capillary GC–MS using a HP6890 GC and a model 5973A MSD with an electron impact ion source and a HP-5 capillary column (30 m x 0.25 mm ID x 0.25 μm film thickness). This protocol was modified from [31].

### Statistical analyses of behavioral and chemical studies

For behavioral assays, owing to sample size, nonparametric procedures were used to assess significant variations in our data. Non-parametric Kruskal–Wallis ANOVAs on ranks for global comparison were performed, followed by corrected Mann–Whitney U tests for multiple comparisons to compare significant differences in aggression indices between treatment groups. For statistical analyses of the chemical profiles, all compounds present above 5% relative levels were used. To assess the similarities of cuticular hydrocarbon profiles, a stepwise discriminant analysis of the relative proportions of the compounds was employed using Statistica 6.0 (StatSoft, Inc., Tulsa, OK, U.S.A.) as in [71]. Prior to analysis, each peak area was standardized according to [72]. Multivariate analysis of variance is known to be resistant to non-normality in the data because of skewness. However, before conducting the analyses, we tested for normality and equality of variance over all groups, both of which were non-significant.

### Microarrays

Individual bees were thawed and dissected under cold RNAlater (Qiagen, Valencia, CA). Abdomens were eviscerated and the cuticles, with the associated fat bodies and oenocytes, were collected. RNA was extracted using an RNeasy kit (Qiagen). RNA was quantified using a Nanodrop 1000 spectrophotometer (Thermo Fischer Scientific, Wilmington, DE) and sample integrity and quality was monitored using agarose gel electrophoresis to confirm the presence of ribosomal RNA bands. 500 ng of RNA/individual was amplified using the Ambion MessageAmp II aRNA Amplification kit (AM1751, Life Technologies, Grand Island, NY). 5 μg of amplified RNA from each sample were labeled independently with Cy3 and Cy5 dyes using the ULS aRNA fluorescent labeling kit (EA-006, Kreatech, Amesterdam, Netherlands). Samples were hybridized to microarrays (two samples/array) in a loop design with dye swaps incorporated, using 24 microarrays for Colony 1 (6 biological replicates, 2 technical replicates per sample) and 16 arrays for Colony 2 (4 biological replicates, 2 technical replicates per sample). Whole genome microarrays (<http://www.ebi.ac.uk/arrayexpress/arrays/A-MEXP-755>) were purchased from the W.M. Keck Center for Functional Genomics at the University of Illinois, Urbana-Champaign. Arrays were scanned using the Axon Genepix 4000B scanner (Molecular Devices, Sunnyvale, CA) using GENEPIX software (Agilent Technologies, Santa Clara, CA).

### Analysis of gene expression

Any spots with an intensity of less than 100 (the background level on the arrays) were removed from the analysis. Also, spots present on less than 12 out of 24 arrays for the first colony and 8 out of the 16 arrays for the second colony were excluded from the data set as well. Expression data was log-transformed and normalized using a mixed-model ANOVA (proc MIXED, SAS, Cary, NC) with the following model:

$$Y=\mu +\text{dye}+\text{block}+{\text{array}}^{*}\text{dye}+{\text{array}}^{*}\text{block}+\varepsilon$$

where Y is expression, dye and block are fixed effects, and array, array*dye and array*block are random effects. Transcripts with significant expression differences between groups were detected by using a mixed-model ANOVA with the model:

Y=μ+treatment+spot+dye+array+ε

where Y represents the residual from the previous model. Treatment, spot and dye are fixed effects and array is a random effect. p-values were corrected for multiple testing using a false discovery rate < 0.01 (proc MULTTEST, SAS). Hierarchical clustering, using the ward method was performed in JMP 9.0.2 (SAS, Cary, NC). Gene ontology analysis was performed using DAVID version 6.7 [73,74] with a cutoff of p <0.05. For all gene ontology (GO) analyses, array transcripts were matched to their *Drosophila* orthologs in Flybase ( http://flybase.org/). All of the array transcripts with *Drosophila* orthologs were used as a background list. Analysis of overlap between the significantly regulated transcripts in our study or between significantly regulated genes in our study and other gene expression studies was performed using Fisher’s Exact Tests (Dr. Oyvind Langsrud, Statistics Norway, <http://www.langsrud.com/fisher.htm>). Common, differentially expressed transcripts or genes between studies were identified with Venny [75]. For overlap comparisons between our study and other studies examining gene expression in honey bees ( [9,38,40]), we converted microarray transcript identifiers (AM numbers) to GB identifiers from the honey bee genome annotation (see BeeBase http://hymenopteragenome.org/beebase/). For comparisons between our study and other studies examining gene expression in fruit flies ( [39,41]), we converted all genes to Flybase identifiers to find common overlap. The array data has been deposited on the ArrayExpress website (E-MEXP-3708).

### Validation of candidate gene expression patterns using quantitative real-time PCR

Worker bees from a fourth source colony were reared as before. Control, saline-injected, and bacteria-injected treatment groups were generated (with 9, 8 and 10 individual bees per treatment group, respectively) as before, but the injected *E. coli* bacteria were collected from an actively growing culture just prior to injection. Bees were collected onto dry ice four hours after treatment and stored at −80°C. RNA was extracted from eviscerated abdomens of individual bees as above. cDNA synthesis and quantitative real-time PCR was performed as in [76]. Expression levels of candidate genes were normalized to actin. Significant differences in expression levels among treatment groups were determined using an ANOVA followed by post-hoc pairwise comparisons with Tukey HSD tests (JMP 9.0.2, SAS, Cary NC). Primer sequences are given in Table 6.

**Table 6:** Primer sequences used to quantify expression of candidate genes from a third biological replicate using quantitative real-time PCR

**Gene name**	**Primer direction**	**Sequence**	**Accession number/ reference**
Lipid Storage Droplet 2	Forward	AATCGTGCTCG CAAAATACC	XM_003249187.1
	Reverse	TTCGCAATAGGT AATGATTTTTCA	
Bubblegum	Forward	TTTTGCAAATCT TGGTGCAA	XM_624222.3
	Reverse	TTCTTCTGGTTT GCCTTCGT	
Pale	Forward	TACTTCGTCGCG GATAGCTT	NM_001011633.1
	Reverse	TACGCGAACGAT GTCTTCAG	
Groucho	Forward	ACTGTGGACAGG AGGATTGG	XM_392425.3
	Reverse	ATGAAGGACCTC GACGTTTG	
Draper	Forward	ATCCTGCCACTG GTTATTGC	XM_624852.2.
	Reverse	AGTCAACTCCCT CCCAACCT	
Apterous	Forward	AGGAAGAGGAAA CCCAAGGA	XM_392622.4
	Reverse	TCTGCGAGAGTTG CTTCAGA	
Pebbled	Forward	CGCTGATACGTCA CCTCAGA	XM_003249649.1
	Reverse	GCGAACTTCTCTC CACGTTC	
Defensin 1	Forward	TGCGCTGCTAACT GTCTCAG	(Evans, [2006])
	Reverse	CGAAACGTTTGTC CCAGAG	NM_001011616.2
Actin	Forward	CCTAGCACCATCC ACCATGAA	(Huising & Flik, 2005)
	Reverse	GAAGCAAGAATTG ACCCACCAA	

Primer sequences were designed in Primer3, v. 4.0 (http://frodo.wi.mit.edu/) or obtained from previous publications.

## Abbreviations

AMPs: Antimicrobial peptides; Bgm: *Bubblegum* gene; CO_2_: Carbon Dioxide; DDC: Dopa-decarboxylase; FDR: False Discovery Rate; GC: Gas chromatography; GO: Gene ontology; *Grh*: Grainy head; Imd: Immune deficiency pathway; *Lsd*: Lipid storage droplet; JAK/STAT: Janus protein kinase (JAK)/Signal transducers and activators of transcription (STAT); JNK: Jun N-terminal Kinase; K W: Kruskal–Wallis; LB media: Luria Broth media; MD: Mahalanobis Distance; MS: Mass Specrometry; QMP: Queen Mandibular Pheromone; RH: Relative humidity; RNA: Ribonucleicacid; Sodh-2: Sorbital dehydrogenase-2; Tps1: Trehalose-6-phospate synthetase 1.

## Competing interests

The authors declare no competing interests.

## Authors’ contributions

FJR performed the treatments, collections, behavioral analyses and chemical analyses. CMG performed the microarray analysis and HLH analyzed the microarray data. FJR and CMG designed the study. FJR, HLH, and CMG wrote the manuscript. All authors read and approved the final manuscript.

## Supplementary Material

Additional file 1 **Table S1.** All (670) significantly, differently expressed transcripts in Colony 1 (FDR<0.01). **Table S2** All (1610) significantly, differently expressed transcripts in Colony 2 (FDR<0.01). **Table S3** The 302 common, significantly, differently expressed transcripts in Colonies 1 and 2 (FDR<0.01) and their log-2 transformed, normalized, relative fold-expression values across treatment groups. Table S3b: p-values (FDR < 0.01) for all treatment pairwise comparisons (Control X Bacteria, Control X Bead, Control X Saline, Bacteria X Bead, Bacteria X Saline, Bead X Saline) for the 302 transcripts significantly regulated in both colonies (see Table S3a). **Table S4** Common, significantly differentially expressed transcripts in treatment groups (control x bacteria, control x bead, control x saline) in both Colonies 1 and 2 (FDR < 0.01). **Table S5** 22 significantly, differentially regulated transcripts regulated by treatment (control x bacteria, control x bead, control x saline) in both Colonies 1 and 2 (FDR < 0.01). **Table S6** Overrepresented GO categories (p < 0.05) amongst the 302 transcripts regulated by treatments in Colonies 1 and 2 (FDR < 0.01). **Table S7** Overrepresented GO categories (p < 0.05) amongst the 22 transcripts regulated by all treatments in Colonies 1 and 2 (FDR < 0.01). **Table S8** The 68 significantly, differently expressed transcripts in bacterial x control treatment groups in common between Colonies 1 and 2 (FDR<0.01). **Table S9** Overrepresented GO categories (p < 0.05) amongst the 68 transcripts regulated by bacterial treatment only in Colonies 1 and 2 (FDR < 0.01). **Table S10** 14 genes that overlap between canonical immune genes (Evans et al., [2006]) and the 239 signficantly, differentially expressed genes in Colonies 1 and 2 (FDR < 0.01). **Table S11** 117 genes that were significantly regulated by Varroa infestation (Alaux et al, [2011]) and immune challenge (this study). **Table S12** 8 genes that were significantly regulated by immune challenge in honey bees (this study) and in Drosophila (De Gregario et al. [2011] and Roxstrom-Linquist et al. [2004]). Click here for file

## References

[B1] Vanengelsdorp D Meixner MD A historical review of managed honey bee populations in Europe and the United States and the factors that may affect them J Invertebr Pathol 2010 103 Suppl 1 S80 S95 19909973 10.1016/j.jip.2009.06.011

[B2] Winston ML The Biology of the Honey Bee 1987 Cambridge: Harvard University Press

[B3] Brown P Cinderella goes to the ball Nature 2001 410 6832 1018 1020 10.1038/35074279 11323633

[B4] Bonasio R Zhang G Ye C Mutti NS Fang X Qin N Donahue G Yang P Li Q Li C Genomic comparison of the ants Camponotus floridanus and Harpegnathos saltator Science 2010 329 5995 1068 1071 10.1126/science.1192428 20798317 PMC3772619

[B5] Gerardo NM Altincicek B Anselme C Atamian H Barribeau SM de Vos M Duncan EJ Evans JD Gabaldon T Ghanim M Immunity and other defenses in pea aphids, Acyrthosiphon pisum Genome Biol 2010 11 2 R21 10.1186/gb-2010-11-2-r21 20178569 PMC2872881

[B6] Smith CD Zimin A Holt C Abouheif E Benton R Cash E Croset V Currie CR Elhaik E Elsik CG Draft genome of the globally widespread and invasive Argentine ant (Linepithema humile) Proc Natl Acad Sci USA 2011 108 14 5673 5678 10.1073/pnas.1008617108 21282631 PMC3078359

[B7] Smith CR Smith CD Robertson HM Helmkampf M Zimin A Yandell M Holt C Hu H Abouheif E Benton R Draft genome of the red harvester ant Pogonomyrmex barbatus Proc Natl Acad Sci USA 2011 108 14 5667 5672 10.1073/pnas.1007901108 21282651 PMC3078412

[B8] Werren JH Richards S Desjardins CA Niehuis O Gadau J Colbourne JK Beukeboom LW Desplan C Elsik CG Grimmelikhuijzen CJ Functional and evolutionary insights from the genomes of three parasitoid Nasonia species Science 2010 327 5963 343 348 10.1126/science.1178028 20075255 PMC2849982

[B9] Evans JD Aronstein K Chen YP Hetru C Imler JL Jiang H Kanost M Thompson GJ Zou Z Hultmark D Immune pathways and defence mechanisms in honey bees Apis mellifera Insect Mol Biol 2006 15 5 645 656 10.1111/j.1365-2583.2006.00682.x 17069638 PMC1847501

[B10] Lemaitre B Hoffmann J The host defense of Drosophila melanogaster Annu Rev Immunol 2007 25 697 743 10.1146/annurev.immunol.25.022106.141615 17201680

[B11] Wilson-Rich N Spivak M Fefferman NH Starks PT Genetic, individual, and group facilitation of disease resistance in insect societies Annu Rev Entomol 2009 54 405 423 10.1146/annurev.ento.53.103106.093301 18793100

[B12] Chen YP Siede R Maramorosh K, Shatkin AJ, Murphy FA Honey Bee Viruses Advances in Virus Research 2007 San Diego, California: Elsevier Academic Press 34 80

[B13] Maori E Lavi S Mozes-Koch R Gantman Y Peretz Y Edelbaum O Tanne E Sela I Isolation and characterization of Israeli acute paralysis virus, a dicistrovirus affecting honeybees in Israel: evidence for diversity due to intra- and inter-species recombination J Gen Virol 2007 88 Pt 12 3428 3438 18024913 10.1099/vir.0.83284-0

[B14] Runckel C Flenniken ML Engel JC Ruby JG Ganem D Andino R DeRisi JL Temporal Analysis of the Honey Bee Microbiome Reveals Four Novel Viruses and Seasonal Prevalence of Known Viruses, Nosema, and Crithidia PLoS One 2011 6 6 e20656 10.1371/journal.pone.0020656 21687739 PMC3110205

[B15] Aronstein KA Murray KD Chalkbrood disease in honey bees J Invertebr Pathol 2010 103 Suppl 1 S20 S29 19909969 10.1016/j.jip.2009.06.018

[B16] Forsgren E European foulbrood in honey bees J Invertebr Pathol 2010 103 Suppl 1 S5 S9 20105559 10.1016/j.jip.2009.06.016

[B17] Genersch E Paenibacillus larvae and American Foulbrood - long since known and still surprising Journal fur Verbrauchershutz und Lebensmittelsicherheit 2008 3 429 434 10.1007/s00003-008-0379-8

[B18] Chen YP Huang ZY Nosema ceranae, a newly identified pathogen of Apis mellifera in the USA and Asia Apidologie 2010 41 3 364 374 10.1051/apido/2010021

[B19] Sammataro D Gerson U Needham G Parasitic mites of honey bees: life history, implications, and impact Annu Rev Entomol 2000 45 519 548 10.1146/annurev.ento.45.1.519 10761588

[B20] Broderick NA Welchman DP Lemaitre B Rolff J, Reynolds S Recognition and response to microbial infection in Drosophila Insect Infection and Immunity: Evolution, Ecology and Mechanisms 2009 Oxford: Oxford University Press 13 33

[B21] Rolff J Reynolds SE Rolff J, Reynolds S Introducing insect infection and immunity Insect Infection and Immunity: Evolution, Ecology and Mechanisms 2009 Oxford:Oxford University Press 1 12

[B22] Schmid-Hempel P Evolutionary ecology of insect immune defenses Annu Rev Entomol 2005 50 529 551 10.1146/annurev.ento.50.071803.130420 15471530

[B23] Ganesan S Aggarwal K Paquette N Silverman N NF-kappa B/Rel Proteins and the Humoral Immune Responses of Drosophila melanogaster Nf-Kb in Health and Disease 2011 349 25 60 10.1007/82_2010_107 PMC3083852 20852987

[B24] Martin P Parkhurst SM Parallels between tissue repair and embryo morphogenesis Development 2004 131 3021 3034 10.1242/dev.01253 15197160

[B25] Dionne MS Schneider DS Models of infectious diseases in the fruit fly Drosophila melanogaster Dis Model Mech 2008 1 1 43 49 10.1242/dmm.000307 19048052 PMC2561979

[B26] Cremer S Armitage SA Schmid-Hempel P Social immunity Curr Biol 2007 17 16 R693 R702 10.1016/j.cub.2007.06.008 17714663

[B27] Simone M Evans JD Spivak M Resin collection and social immunity in honey bees Evolution 2009 63 11 3016 3022 10.1111/j.1558-5646.2009.00772.x 19619221

[B28] Rueppell O Hayworth MK Ross NP Altruistic self-removal of health-compromised honey bee workers from their hive J Evol Biol 2010 23 7 1538 1546 10.1111/j.1420-9101.2010.02022.x 20500363

[B29] Hassanein MH The Influence of Infection with Nosema-Apis on the Activities and Longevity of the Worker Honeybee Ann Appl Biol 1953 40 2 418 423 10.1111/j.1744-7348.1953.tb01093.x

[B30] Traniello JF Rosengaus RB Savoie K The development of immunity in a social insect: evidence for the group facilitation of disease resistance Proc Natl Acad Sci USA 2002 99 10 6838 6842 10.1073/pnas.102176599 12011442 PMC124490

[B31] Richard FJ Aubert A Grozinger CM Modulation of social interactions by immune stimulation in honey bee, Apis mellifera, workers BMC Biol 2008 6 50 10.1186/1741-7007-6-50 19014614 PMC2596086

[B32] Fan Y Zurek L Dykstra MJ Schal C Hydrocarbon synthesis by enzymatically dissociated oenocytes of the abdominal integument of the German Cockroach, Blattella germanica Naturwissenschaften 2003 90 3 121 126 12649753 10.1007/s00114-003-0402-y

[B33] Howard RW Blomquist GJ Ecological, behavioral, and biochemical aspects of insect hydrocarbons Annu Rev Entomol 2005 50 371 393 10.1146/annurev.ento.50.071803.130359 15355247

[B34] Blomquist GJ Bagneres AG Insect hydrocarbons: biology, biochemistry and chemical ecology 2010 Cambridge: Cambridge University Press

[B35] Fan YL Richard FJ Rouf N Grozinger CM Effects of queen mandibular pheromone on nestmate recognition in worker honeybees, Apis mellifera Anim Behav 2010 79 3 649 656 10.1016/j.anbehav.2009.12.013

[B36] Salvy M Martin C Bagneres AG Provost E Roux M Le Conte Y Clement JL Modifications of the cuticular hydrocarbon profile of Apis mellifera worker bees in the presence of the ectoparasitic mite Varroa jacobsoni in brood cells Parasitology 2001 122 Pt 2 145 159 11272645 10.1017/s0031182001007181

[B37] Kulincevic JM Stairs GR Rothenbuhler WC A disease of the honey bee causing behavioral changes and mortality J Invertebr Pathol 1969 14 1 13 17 10.1016/0022-2011(69)90004-4 5816958

[B38] Alaux C Dantec C Parrinello H Le Conte Y Nutrigenomics in honey bees: digital gene expression analysis of pollen's nutritive effects on healthy and varroa-parasitized bees BMC Genomics 2011 12 496 10.1186/1471-2164-12-496 21985689 PMC3209670

[B39] De Gregorio E Spellman PT Rubin GM Lemaitre B Genome-wide analysis of the Drosophila immune response by using oligonucleotide microarrays Proc Natl Acad Sci USA 2001 98 22 12590 12595 10.1073/pnas.221458698 11606746 PMC60098

[B40] Navajas M Migeon A Alaux C Martin-Magniette M Robinson G Evans J Cros-Arteil S Crauser D Le Conte Y Differential gene expression of the honey bee Apis mellifera associated with Varroa destructor infection BMC Genomics 2008 9 301 10.1186/1471-2164-9-301 18578863 PMC2447852

[B41] Roxstrom-Lindquist K Terenius O Faye I Parasite-specific immune response in adult Drosophila melanogaster: a genomic study EMBO Rep 2004 5 2 207 212 10.1038/sj.embor.7400073 14749722 PMC1298984

[B42] Decanini LI Collins AM Evans JD Variation and heritability in immune gene expression by diseased honeybees J Hered 2007 98 3 195 201 10.1093/jhered/esm008 17404328

[B43] Evans JD Transcriptional immune responses by honey bee larvae during invasion by the bacterial pathogen, Paenibacillus larvae J Invertebr Pathol 2004 85 2 105 111 10.1016/j.jip.2004.02.004 15050840

[B44] Wittkopp PJ Beldade P Development and evolution of insect pigmentation: genetic mechanisms and the potential consequences of pleiotropy Semin Cell Dev Biol 2009 20 1 65 71 10.1016/j.semcdb.2008.10.002 18977308

[B45] Munoz-Descalzo S Terol J Paricio N Cabut, aC(2)H(2) zinc finger transcription factor, is required during Drosophila dorsal closure downstream of JNK signaling Dev Biol 2005 287 1 168 179 10.1016/j.ydbio.2005.08.048 16198331

[B46] Kambris Z Brun S Jang IH Nam HJ Romeo Y Takahashi K Lee WJ Ueda R Lemaitre B Drosophila immunity: a large-scale in vivo RNAi screen identifies five serine proteases required for Toll activation Curr Biol 2006 16 8 808 813 10.1016/j.cub.2006.03.020 16631589

[B47] Blomquist GJ Blomquist GJ, Bagneres AG Biosynthesis of cuticular hydrocarbons Insect hydrocarbons: biology, biochemistry and chemical ecology 2010 Cambridge: Cambridge University Press 35 52

[B48] Min KT Benzer S Preventing neurodegeneration in the Drosophila mutant bubblegum Science 1999 284 5422 1985 1988 10.1126/science.284.5422.1985 10373116

[B49] Teixeira L Rabouille C Rorth P Ephrussi A Vanzo NF Drosophila perilipin/ADRP homologue Lsd2 regulates lipid metabolism Mech Dev 2003 120 9 1071 1081 10.1016/S0925-4773(03)00158-8 14550535

[B50] Beller M Riedel D Jansch L Dieterich G Wehland J Jackle H Kuhnlein RP Characterization of the Drosophila lipid droplet subproteome Mol Cell Proteomics 2006 5 6 1082 1094 10.1074/mcp.M600011-MCP200 16543254

[B51] Ayres JS Schneider DS The Role of Anorexia in Resistance and Tolerance to Infections in Drosophila PLoS Biol 2009 7 7 e1000150 10.1371/journal.pbio.1000150 19597539 PMC2701602

[B52] Luque T Hjelmqvist L Marfany G Danielsson O El-Ahmad M Persson B Jornvall H Gonzalez-Duarte R Sorbitol dehydrogenase of Drosophila - Gene, protein, and expression data show a two-gene system J Biol Chem 1998 273 51 34293 34301 10.1074/jbc.273.51.34293 9852094

[B53] Chen QF Haddad GG Role of trehalose phosphate synthase and trehalose during hypoxia: from flies to mammals J Exp Biol 2004 207 18 3125 3129 10.1242/jeb.01133 15299033

[B54] Scherfer C Tang H Kambris Z Lhocine N Hashimoto C Lemaitre B Drosophila Serpin-28D regulates hemolymph phenoloxidase activity and adult pigmentation Dev Biol 2008 323 2 189 196 10.1016/j.ydbio.2008.08.030 18801354

[B55] Dani FR Jones GR Corsi S Beard R Pradella D Turillazzi S Nestmate recognition cues in the honey bee: differential importance of cuticular alkanes and alkenes Chem Senses 2005 30 6 477 489 10.1093/chemse/bji040 15917370

[B56] Hamilton C Lejeune BT Rosengaus RB Trophallaxis and prophylaxis: social immunity in the carpenter ant Camponotus pennsylvanicus Biol Lett 2011 7 1 89 92 10.1098/rsbl.2010.0466 20591850 PMC3030872

[B57] Aubert A Richard FJ Social management of LPS-induced inflammation in Formica polyctena ants Brain Behav Immun 2008 22 6 833 837 10.1016/j.bbi.2008.01.010 18331785

[B58] Hughes WOH Eilenberg J Boomsma JJ Trade-offs in group living: transmission and disease resistance in leaf-cutting ants Proc R Soc Lond B 2002 269 1811 1819 10.1098/rspb.2002.2113 PMC1691100 12350269

[B59] Traniello JFA Rosengaus RB Savoie K The development of immunity in a social evidence for the group facilitation of disease resistance Proc Natl Acad Sci USA 2002 99 6838 6842 10.1073/pnas.102176599 12011442 PMC124490

[B60] Zecchini V Brennan K Martinez-Arias A An activity of Notch regulates JNK signalling and affects dorsal closure in Drosophila Curr Biol 1999 9 9 460 469 10.1016/S0960-9822(99)80211-5 10322111

[B61] Arrese EL Soulages JL Insect Fat Body: Energy, Metabolism, and Regulation Annu Rev Entomol 2010 55 207 225 10.1146/annurev-ento-112408-085356 19725772 PMC3075550

[B62] Bischoff V Vignal C Duvic B Boneca IG Hoffmann JA Royet J Downregulation of the Drosophila immune response by peptidoglycan-recognition proteins SC1 and SC2 PLoS Pathog 2006 2 2 e14 10.1371/journal.ppat.0020014 16518472 PMC1383489

[B63] Schluns H Crozier RH Relish regulates expression of antimicrobial peptide genes in the honeybee, Apis mellifera, shown by RNA interference Insect Mol Biol 2007 16 6 753 759 10.1111/j.1365-2583.2007.00768.x 18093004

[B64] Dade HA Anatomy and Dissection of the Honeybee 1994 Cardiff: International Bee Research Association

[B65] Gutierrez E Wiggins D Fielding B Gould AP Specialized hepatocyte-like cells regulate Drosophila lipid metabolism Nature 2007 445 7125 275 280 10.1038/nature05382 17136098

[B66] Mashek DG Li LO Coleman RA Long-chain acyl-CoA synthetases and fatty acid channeling Future Lipidology 2007 2 4 465 476 10.2217/17460875.2.4.465 20354580 PMC2846691

[B67] Pei ZT Oey NA Zuidervaart MM Jia ZZ Li YY Steinberg SJ Smith KD Watkins PA The acyl-CoA synthetase "bubblegum" (lipidosin) - Further characterization and role in neuronal fatty acid beta-oxidation J Biol Chem 2003 278 47 47070 47078 10.1074/jbc.M310075200 12975357

[B68] Grozinger CM Sharabash NM Whitfield CW Robinson GE Pheromone-mediated gene expression in the honey bee brain Proc Natl Acad Sci USA 2003 100 Suppl 2 14519 14525 14573707 10.1073/pnas.2335884100 PMC304112

[B69] Hoover SE Keeling CI Winston ML Slessor KN The effect of queen pheromones on worker honey bee ovary development Naturwissenschaften 2003 90 10 477 480 10.1007/s00114-003-0462-z 14564409

[B70] Yang X Cox-Foster DL Impact of an ectoparasite on the immunity and pathology of an invertebrate: evidence for host immunosuppression and viral amplification Proc Natl Acad Sci USA 2005 102 21 7470 7475 10.1073/pnas.0501860102 15897457 PMC1140434

[B71] Richard FJ Poulsen M Drijfhout F Jones G Boomsma JJ Specificity in chemical profiles of workers, brood and mutualistic fungi in Atta, Acromyrmex, and Sericomyrmex fungus-growing ants J Chem Ecol 2007 33 12 2281 2292 10.1007/s10886-007-9385-z 18040743

[B72] Reyment RA Compositional data analysis Terra Nova 1989 1 1 29 34 10.1111/j.1365-3121.1989.tb00322.x

[B73] Dennis G Jr Sherman BT Hosack DA Yang J Gao W Lane HC Lempicki RA DAVID: Database for Annotation, Visualization, and Integrated Discovery Genome Biol 2003 4 5 P3 10.1186/gb-2003-4-5-p3 12734009 PMC193660

[B74] da Huang W Sherman BT Lempicki RA Systematic and integrative analysis of large gene lists using DAVID bioinformatics resources Nat Protoc 2009 4 1 44 57 19131956 10.1038/nprot.2008.211

[B75] VENNY An interactive tool for comparing lists with Venn Diagramshttp://bioinfogp.cnb.csic.es/tools/venny/index.html

[B76] Fussnecker BL McKenzie AM Grozinger CM cGMP modulates responses to queen mandibular pheromone in worker honey bees J Comp Physiol A Neuroethol Sens Neural Behav Physiol 2011 197 9 939 948 10.1007/s00359-011-0654-5 21626397 PMC3705726

